# Low potassium and high sodium intakes: a double health threat to Cape Verdeans

**DOI:** 10.1186/s12889-018-5911-x

**Published:** 2018-08-09

**Authors:** Daniela Alves, Zélia Santos, Miguel Amado, Isabel Craveiro, António Pedro Delgado, Artur Correia, Luzia Gonçalves

**Affiliations:** 1 Instituto de Higiene e Medicina Tropical - Universidade Nova de Lisboa, IHMT-UNL, Rua da Junqueira 100, 1349-008 Lisbon, Portugal; 2 Global Health and Tropical Medicine, GHTM, IHMT-UNL, Lisbon, Portugal; 3 Civil Enginnering Research and Inovation for Sustainability, CERis, Instituto Superior Técnico, Universidade de Lisboa, Lisboa, Portugal; 4 Direção Nacional da Saúde, Ministério da Saúde, Praia, Cape Verde; 5 Comité de Coordenação Combate à SIDA, Ministério da Saúde, Praia, Cape Verde; 6 Centro de Estatística e Aplicações da Universidade de Lisboa, Lisbon, Portugal

**Keywords:** Potassium, Sodium, Cardiovascular diseases, Hypertension, Overweight, Obesity, Formal and informal areas, Cape Verde, Sub-Saharan Africa

## Abstract

**Background:**

Cape Verde presents a high rate of cardiovascular diseases. Low potassium and high sodium intakes are related to cardiovascular diseases. However, studies regarding these two micronutrients continue to be rare in African urban settings. This work aims to estimate potassium and sodium intakes and to analyse the self-reported salt intake by gender and by type of urban area in the city of Praia – the capital of Cape Verde.

**Methods:**

In the first stage (*n* = 1912), an intra-urban study was designed in two types of urban areas (formal and informal), using a sampling strategy based on random selection of geographical coordinates, in order to apply a questionnaire. In a second stage, a 24-h dietary recall and anthropometric measurements were performed by local nutritionists. Potassium and sodium intakes were estimated for 599 participants (149 men and 450 women). Non-parametric methods (including quantile regression) were used in the statistical analysis.

**Results:**

In informal areas, a higher percentage of women reported having hypertension (31.0%) compared to formal areas (19.7%). Based on 24-h dietary recall, median potassium intake for men was 2924.2 mg/day and for women and 2562.6 mg/day. Almost 70.0% of men and 80.0% of women ingested less than the recommended 3510 mg/day of potassium. In informal areas, men and women presented high medians of sodium intakes compared to formal areas (men: 4131.2 vs 3014.6 mg/day and women: 3243.4 vs 2522.4 mg/day). On the other hand, the percentage of participants exceeding 2000 mg/day for sodium was high (≥70.8%), even for participants that self-reported low-salt intake.

Quantile regression models revealed effects of the type of urban area and gender in the potassium and sodium intakes, at least, in some quartiles, accounting for age, academic qualifications, and professional situation.

**Conclusions:**

A low potassium intake and a high sodium intake were found in Praia. Thus, efficient health education campaigns and health promotion are needed and should be tailored considering gender and urban areas.

## Background

Non-communicable chronic diseases have been increasing steadily both in developed countries as well as in low- and middle-income countries [1]. Dietary intake assessments in low- and middle-income countries are crucial to providing detailed information regarding micronutrients and their effects on health [2]. High levels of sodium have been associated with elevated blood pressure and poor cardiovascular health [3–10], while other studies have suggested that potassium intake may have a protective effect against strokes and cardiovascular disease [6, 11–13].

According to the World Health Organization (WHO) recommendations, daily sodium intake should be less than 2000 mg (5000 mg of salt) [14]. A minimum potassium daily intake of 3510 mg has been recommended [15].

Some authors have highlighted the role of socioeconomic inequalities to design interventions aiming to promote a healthy diet, such as the reduction of salt intake [16, 17].

Africa has been undergoing a rapid process of urbanization (marked by lifestyles changes such as physical inactivity, high salt diets, fried food, high fat food, alcohol, and tobacco) that contributes to an increase in obesity, hypertension and cardiovascular diseases [18–20].

There is a lack of studies on potassium and sodium intake in African countries, and the few existing estimates are uncertain [6]. According to a systematic review published in 2016 focused on salt intake in sub-Saharan Africa (a study which did not include Cape Verde), only six studies considered gender differences in urban settings, the most recent being published in 2001 [21]. Because publications on potassium and sodium intake in African cities are rare, there is an important gap to fill, particularly with regard to potential differences by gender and urban area.

Depending on the type of urban setting under investigation, some differences in food consumption habits are expected between the neighbourhoods of a city [22, 23]. Although living in a disadvantaged neighbourhood has been associated with a poorer diet [24, 25], some studies have shown the opposite may be true [22, 23]. This incongruence seems to suggest that intra-urban variations are locally dependent, with each city presenting a different pattern.

Cape Verde is an archipelago island nation in sub-Saharan Africa, located in the central Atlantic Ocean, which is composed of ten islands. The population of Santiago, the largest island, is concentrated in the city of Praia (the capital of Cape Verde) [26]. In this medium-income country, 69.0% of all deaths are caused by non-communicable diseases, of which 35.0% are due to cardiovascular diseases [27].

Dope et al. [28] show that Cape Verdean families consume a lower proportion of fruits and vegetables than recommended by WHO, an important source of potassium. As dietary habits in Cape Verde has changed, a decrease in the consumption of traditional products (e.g. manioc, sweet potatoes or corn) and an increase in the consumption of processed foods and oils suggest a nutritional transition stage in this country [28, 29]. These results, along with empirical knowledge and unpublished studies, suggest a high sodium intake in the Cape Verdean population. To our knowledge, there are no published studies on the intake of potassium and sodium in Cape Verde.

This study aims to estimate the potassium and sodium intakes and to analyse the self-perception of salt intake of men and women living in formal and informal areas of the city of Praia.

## Methods

This work is part of a research project: “UPHI-STAT: Urban Planning and Health Inequities – moving from macro to micro statistics”. This intra-urban study was originally conducted in the city of Praia, in three neighbourhoods - Plateau (formal area), in part of Vila Nova (informal area) and Palmarejo (formal and informal areas). Formal urban areas include elements of urban planning and are equipped with public services, infrastructures, and green spaces [30–32]. The informal urban type is characterized by an irregular matrix composed of clandestine and randomly exposed buildings without a planning model [30–32].

Data collection occurred between January and October 2014. A random sampling strategy based on geographical coordinates of private households was used to select in each household one adult (≥ 18 years), living at least six months in the neighbourhood. Methodological aspects of the fieldwork were described in more detail in the previous study [30]. Briefly, the first stage involved a random sample size of 1912 adults who answered the UPHI-STAT questionnaire, applied by trained local interviewers [30]. A pre-test of the questionnaire was previously carried out [30]. Some variables from the UPHI-STAT questionnaire are explored in this work, namely sociodemographic characteristics, self-reported chronic diseases (e.g., self-reported hypertension), and frequency of fruits and vegetables consumption. Self-reported chronic diseases and hypertension were ascertained with the questions: “Do you have any chronic disease diagnosed by a health professional?” and if yes, “Do you have hypertension?” (Yes/No). Self-reported salt intake was explored by this question: “How do you classify the amount of salt you consume regularly?”, with answers: “do not use salt”, “low”, “normal” and “enough/add salt”.

In the second stage, all participants of the first stage were invited to visit local nutritionists and 599 participants consented and undergo a nutritional status evaluation [30]. This evaluation included a 24-h dietary recall (24HDR), collecting information on food and beverage consumption during the previous 24 h. Additionally, local nutritionists asked about dietary habits during weekends or holidays. For the 24HDR application, standard household measuring utensils and validated food photographs were used to better quantify food portions. This method was conducted to determine the potassium and sodium intakes from food sources alone. Following the practice conducted in Cape Verde by local nutritionists, nutritional data from 24HDR were obtained using the Portuguese Food Composition Table [33] and, where necessary, additional information was obtained from the West African Food Composition Table, developed by the Food and Agriculture Organization [34]. The values of sodium and potassium intake were then compared to the WHO recommendations [14, 15]. Nutritional status was assessed, among others measures, from Body Mass Index (BMI) [35] and waist circumference (WC) [36]. The categories associated with BMI ranges for adults are: underweight (< 18.5 kg/m2); normal weight (18.5–24.9 kg/m2); overweight (25.0–29.9 kg/m2) and obesity (≥30.0 kg/m2). The cardiometabolic risk was based on the WC by gender: increased risk (men: > 94 cm, women: > 80 cm) and high increased risk (men: > 102 cm, women: > 88 cm) [36]. In this stage, 149 men (47 in the formal and 102 in the informal areas) and 450 women (153 in the formal and 297 in the informal areas) were included in this analysis. All participants provided written informed consent and this study was approved by ethical committees for Ethics in Research for Health (doc.N.52/2013), Cape Verde, and Ethics Council of IHMT (doc n°.24–2013-PI), Portugal.

### Statistical analyses

Statistical analysis was performed using SPSS version 22.0 and R Statistical Software. Frequency tables were made for qualitative variables. Median and interquartile range (IQR) were used for asymmetric quantitative or ordinal variables. In order to decide between parametric or non-parametric tests, normality tests of Kolmogorov-Smirnov and Shapiro-Wilk were used, as well as the Levene test for homogeneity of variances. Since none of the studied variables fulfilled these assumptions, to compare by the two types of areas and by gender, Mann–Whitney U tests were used. Differences between proportions were performed with Chi-square test or Fisher’s exact test. Additionally, a multivariate analysis was performed to explore if gender and type of urban area explain differences in potassium and sodium intakes, considering some confounding factors (e.g. age, academic qualifications and professional situation). Instead of traditional linear regression, quantile regression models were explored for each micronutrient. This choice was due to the failure of the normality assumption and some outliers in sodium and potassium intakes (dependent variables). Quantile regression is more robust and provides possible heterogeneous effects of explanatory variables at different parts or points (e.g., quartiles) of the conditional distribution of the dependent variable. We will only present the results to the first quartile (Q1), second quartile (Q2 - median) and third quartile (Q3), in order to illustrate the effects of covariates in lower, intermediate and higher values of sodium and potassium intakes [37, 38].

## Results

The sociodemographic characteristics, self-reported chronic diseases (e.g., hypertension) and nutritional status of men and women, respectively in the two types of urban areas are presented in Tables 1 and 2.

**Table 1:** Sociodemographic characteristics, some aspects related to self-reported health status and nutritional status of men in urban areas

Variables/Men	Formal (*n* = 47)	Informal (*n* = 102)	Total (*n* = 149)	*P*
Age, median (IQR)	27.0 (22.0–44.25)	35.5 (26.8–45.0)	33.5 (25.0–45.0)	**0.049**
Academic qualifications, *n* (%)				**0.002**
None and preschool	5 (10.6)	8 (7.8)	13 (8.7)	
Primary	4 (8.5)	28 (27.5)	32 (21.5)	
Secondary	21 (44.7)	53 (52.0)	74 (49.7)	
High school	17 (36.2)	13 (12.7)	30 (20.1)	
Professional Status, *n* (%)				**0.032**
Unemployed	5 (10.6)	28 (27.5)	33 (22.1)	
Employed	42 (89.4)	74 (72.5)	116 (77.9)	
Chronic disease, *n* (%)	3 (6.5)	24 (23.5)	27 (18.2)	**0.020**
Hypertension, *n* (%)	2 (4.3)	16 (15.7)	18 (12.2)	0.059
BMI (Kg/m^2^), median (IQR)	22.9 (20.6–26.4)	23.9 (21.2–27.7)	23.7 (21.1–27.0)	0.321
BMI classification, *n* (%)				0.857
Underweight	4 (8.5)	7 (6.9)	11 (7.4)	
Normal weight	27 (57.4)	56 (54.9)	83 (55.7)	
Overweight and obesity	16 (34.0)	39 (38.2)	55 (36.9)	
WC (cm), median (IQR)	81.0 (74.5–89.0)	85.00 (78.0–94.0)	83.5 (76.5–91.0)	0.065
Metabolic Risk, *n* (%)				
No risk	40 (85.1)	76 (74.5)	116 (77.9)	0.274
Increased risk	2 (4.3)	12 (11.8)	14 (9.4)	
High increased risk	5 (10.6)	14 (13.7)	19 (12.8)	

*IQR* interquartile range, *BMI* body mass index, *WC* waist circumference, *P*, p-value - Mann Whitney U-test or Chi-square

Values that are statistically significant at the 5% level are marked in bold

**Table 2:** Sociodemographic characteristics, some aspects related to self-reported health status and nutritional status of women in urban areas

Variables/Women	Formal (*n* = 153)	Informal (*n* = 297)	Total (*n* = 450)	*P*
Age, median (IQR)	36.00 (28.00–48.00)	40.00 (28.00–54.50)	38.00 (28.00–53.00)	0.056
Academic qualifications, *n* (%)				**0.008**
None and preschool	23 (15.3)	70 (23.7)	93 (20.9)	
Primary	41 (27.3)	100 (33.9)	141 (31.7)	
Secondary	50 (33.3)	85 (28.8)	135 (30.3)	
High school	36 (24.0)	40 (13.6)	76 (17.1)	
Professional Status, *n* (%)				**0.001**
Unemployed	25 (16.4)	94 (31.6)	119 (26.5)	
Employed	127 (83.6)	203 (68.4)	330 (73.5)	
Chronic disease, *n* (%)	49 (32.2)	116 (39.5)	165 (37.0)	0.148
Hypertension, *n* (%)	30 (19.7)	92 (31.0)	122 (27.4)	**0.010**
BMI (Kg/m^2^), median (IQR)	26.0 (22.1–30.2)	26.7 (22.8–31.1)	26.5 (22.6–30.9)	0.315
BMI classification, *n* (%)				0.392
Underweight	12 (7.9)	29 (9.9)	41 (9.2)	
Normal weight	52 (34.2)	83 (28.2)	135 (30.3)	
Overweight and obesity	88 (57.9)	182 (61.9)	270 (60.5)	
WC (cm), median (IQR)	89.0 (77.5–98.0)	92.0 (80.0–102.0)	91.00 (79.0–101.0)	**0.047**
Metabolic Risk, *n* (%)				0.222
No risk	45 (29.8)	67 (23.0)	112 (25.3)	
Increased risk	25 (16.6)	44 (15.1)	69 (15.6)	
High increased risk	81 (53.6)	180 (61.9)	261 (59.0)	

*IQR* interquartile range, *BMI* body mass index, *WC* waist circumference, *P* p-value - Mann Whitney U-test or Chi-square

Values that are statistically significant at the 5% level are marked in bold

### Sociodemographic characteristics

Over 60.0% of participants reported having primary or secondary education levels, with significant differences by urban area being seen for both men and women, with the residents of informal areas reporting less education. By area, unemployment rates were significantly higher in informal areas for both men and women. Gender differences were found for age (*P* = .006, data not show) and for academic qualifications (*P* < .001, data not shown). Women are older and have lower academic qualifications compared to men.

### Self-reported chronic diseases

Regarding self-reported chronic diseases, 18.2% of men (Table 1) reported being diagnosed with a chronic disease, with a significant difference between areas (formal: 6.5% vs. informal: 23.5%). According to Table 1, 12.2% of men reported having hypertension with no difference between urban areas.

For women (Table 2), 37.0% reported suffering from a chronic disease. Comparing the percentage of women who reported hypertension, a significant difference was observed between formal (19.7%) and informal areas (31.0%). Data analysis has shown that women reported chronic diseases, particularly hypertension, more frequently than men (*P* < .001, data not shown).

### Nutritional status

With regards to the BMI of men (Table 1), the median was 23.7 kg/m^2^, with 36.9% qualifying as overweight or obese. For women (Table 2), the median of the BMI was higher, 26.5 kg/m^2^, with 60.5% qualifying as overweight or obese. The median WC was 83.5 cm for men and 91.0 cm for women. Medians were slightly higher in informal urban areas, although only a borderline significant difference was found for women (Table 2). More than three-quarter of men (77.9%) and about one-quarter (25.3%) of women did not present cardiometabolic risk according to cut-off values suggested by WHO. Cardiometabolic risk did not differ significantly by urban areas. Compared to men, women have a worse nutritional status, higher proportions of overweight or obesity, and higher cardiometabolic risk, with statistical significance (*P* < .001, data not shown).

Tables 3 and 4 describe the frequency of fruits and vegetables consumption, self-reported salt intake, sodium and potassium intakes, and the comparison with the daily recommended intakes, respectively, for men and women by urban areas.

**Table 3:** Fruits and vegetables, self-reported salt intake, potassium and sodium intakes and dietary recommendations for men by urban areas

Variable/Areas	Formal (*n* = 47)	Informal (*n* = 102)	Total (*n* = 149)	*P*
Men				
Fruits, *n* (%)				0.291^c^
Daily	22 (46.8)	58 (56.9)	80 (53.7)	
Others	25 (53.2)	44 (43.1)	69 (46.3)	
Vegetables, *n* (%)				0.849^c^
Daily	13 (27.7)	30 (29.4)	43 (28.9)	
Others	34 (72.3)	72 (70.6)	106 (71.1)	
K (mg/day), median (IQR)	2628.5 (1922.7–3579.0)	3067.9 (2371.0–3827.0)	2924.2 (2208.9–3726.2)	0.178^b^
Recommendation K intake, *n* (%)				0.447^c^
≥ 3510 mg/day	12 (25.5)	33 (32.4)	45 (30.2)	
< 3510 mg/day	35 (74.5)	69 (67.6)	104 (69.8)	
Self-reported SALT intake, *n* (%)				0.461^a^
Not consume	0 (0.0)	1 (1.0)	1 (0.7)	
Low	21 (44.7)	35 (35.0)	56 (38.1)	
Normal	26 (55.3)	60 (60.0)	86 (58.5)	
Enough/ Add salt	0 (0.0)	4 (4.0)	4 (2.7)	
Na (mg/day), median (IQR)	3014.6 (1907.7–4453.0)	4131.2 (2522.2–5632.0)	3707.4 (2308.6–5219.7)	**0.013** ^b^
Recommendation Na (mg/day), *n* (%)				0.278^c^
< 2000	12 (25.5)	18 (17.6)	30 (20.1)	
≥ 2000	35 (74.5)	84 (82.4)	119 (79.9)	

*K* Potassium, *Na* sodium, *IQR* interquartile range, *P* p-value; ^a^Fisher exact test; ^b^Mann Whitney U-test; ^c^Chi-square

Values that are statistically significant at the 5% level are marked in bold

**Table 4:** Fruits and vegetables, self-reported salt intake, potassium and sodium intakes and dietary recommendations for women by urban areas

Variable/Areas	Formal (*n* = 153)	Informal (*n* = 297)	Total (*n* = 450)	*P*
Women				
Fruits, *n* (%)				0.310^c^
Daily	87 (56.9)	184 (62.0)	271 (60.2)	
Others	66 (43.1)	113 (38.0)	179 (39.8)	
Vegetables, *n* (%)				0.604^c^
Daily	52 (34.2)	110 (37.0)	162 (36.1)	
Others	100 (65.8)	187 (63.0)	287 (63.9)	
K (mg/day), median (IQR)	2453.6 (1801.4–3176.1)	2592.3 (1877.1–3380.5)	2562.6 (1838.0–3321.9)	0.298^b^
Recommendation K intake, *n* (%)				0.066^c^
≥ 3510 mg/day	24 (15.7)	69 (23.3)	93 (20.7)	
< 3510 mg/day	129 (84.3)	227 (76.7)	356 (79.3)	
Self-reported SALT intake, *n* (%)				0.262^a^
Not consume	6 (3.9)	4 (1.3)	10 (2.2)	
Low	81 (52.9)	148 (49.8)	229 (50.9)	
Normal	64 (41.8)	141 (47.5)	205 (45.6)	
Enough/Add salt	2 (1.3)	4 (1.3)	6 (1.3)	
Na (mg/day), median (IQR)	2522.4 (1638.2–4124.2)	3243.4 (1950.0–5304.1)	3019.8 (1786.8–4745.7)	**0.001** ^b^
Recommendation Na (mg/day), *n* (%)				**0.030** ^c^
< 2000	55 (35.9)	77 (26.0)	132 (29.4)	
≥ 2000	98 (64.1)	219 (74.0)	317 (70.6)	

*K* Potassium, *Na* sodium, *IQR* interquartile range, *P*, p-value; ^a^Fisher exact test; ^b^Mann Whitney U-test; ^c^Chi-square

Values that are statistically significant at the 5% level are marked in bold

### Fruits and vegetables consumption

Most men (Table 3) consumed fruit on a daily basis (53.7%), but 71.1% reported not eating vegetables on a daily basis, with no significant differences by urban area. Women faired only slightly better (Table 4), with 60.2% reporting a daily intake of fruit, and a high non-daily consumption of vegetables (63.9%), without differences by urban area.

### Potassium intake

When comparing by urban area, no significant differences were found in terms of potassium intake for both men (Table 3) and women (Table 4). However, there was a significant difference by gender (*P* = .003, data not shown). For men (Table 3), the median daily potassium intake was 2924.2 mg/day (IQR: 2208.9–3726.2 mg/day), with 69.8% of men not meeting the recommended daily potassium intake (≥ 3510 mg/day). For women (Table 4), the median potassium intake was 2562.6 mg/day (IQR: 1838.0–3321.9 mg/day), with 79.3% of women ingested less than the recommended 3510 mg/day of potassium. A significant difference between men and women in terms of the daily potassium recommendation was also found (*P* = .017, data not shown).

### Self-reported salt intake

Regarding self-reported salt intake, 96.2% of men reported a “low” or “normal” consumption (Table 3) of salt. For women, 50.9% reported “low”, and 45.6% reported a “normal” salt intake (Table 4). Only 2.7% of men and 1.3% of women reported “enough” consumption or “always add salt to meals”. A significant difference in terms of self-reported salt intake by gender was found (*P* = .013, data not shown).

### Sodium intake

Sodium intake varied significantly by urban areas, with higher values in informal areas, for both men and women. For men (Table 3), the median value for sodium intake was 3014.6 mg/day (IQR: 1907.7–4453.0 mg/day) in formal areas and 4131.2 mg/day (IQR: 2522.2–5632.0 mg/day) in informal areas. While guidelines recommend that daily sodium intake should not exceed 2000 mg/day, 79.9% of respondents in both urban areas exceeded this value. Furthermore, 20.6% of men in the informal areas presented a daily intake of above 6000 mg/day (data not shown). For women (Table 4) living in formal areas, the median sodium intake was statistically lower (2522.4 mg/day (IQR: 1638.2–4124.2 mg/day)) than the women living in informal areas (3243.4 mg/day (IQR: 1950.0–5304.1 mg/day)). With regards to exceeding the daily sodium intake recommendation, more women from the informal areas (74.0%) exceeded these recommendations compared to women from the formal areas (64.1%, *P* = .030). A percentage of 19.3% for women living in informal and 6.5% living in formal areas presented a sodium intake higher to 6000 mg/day (data not shown). Gender differences were also observed in daily sodium intake, with higher values for men (*P* = .006, data not shown).

### Sodium intake according to self-reported salt intake categories

Sodium intake and exceeding the daily recommendation according to self-reported salt intake (not consume, low, normal or enough/add salt) are presented in Table 5. No significant differences were found by gender and urban areas. Thus, these analyses are presented for the total sample. Participants who reported low salt intake had a median sodium intake of 3158.7 mg/day (IQR: 1830.9–5208.9 mg/day). Sodium intake based on 24HDR did not vary among the four categories of self-reported salt intake by participants. Moreover, the percentage of participants exceeding 2000 mg/day for sodium was high (≥70.8%) in all categories considered for self-reported salt intake.

**Table 5:** Sodium intake and percentage of participants exceeding the current recommendation for sodium, according to self-reported salt intake categories

	Self-reported salt intake categories				
	Not consume (*n* = 11)	Low (*n* = 284)	Normal (*n* = 291)	Enough/Add salt (*n* = 10)	*P*
Na (mg/day), median (IQR)	2408.0 (1948.4–4006.8)	3158.7 (1830.9–5208.9)	3179.8 (1980.9–4749.40)	2613.7 (2212.9–5020.7)	0.647^a^
≥2000 mg/day Na, n (%)	8 (72.7)	201 (70.8)	218 (74.9)	8 (80.0)	0.710^b^

*Na* sodium, *IQR* interquartile range, *P* *p*-value; ^a^Kruskal Wallis test; ^b^Fisher exact test

### Multivariate analysis for the potassium and sodium intakes

An additional statistical analysis was performed to explore if potassium and sodium (mg/day) intakes were affected by gender and by type of urban area when accounting for potential confounding variables. Table 6 presents of the coefficient sign and *p*-values for quantile regression models, namely for Q1, Q2 (median), and Q3.

**Table 6:** Quantile regression for potassium and sodium intakes, considering socioeconomic variables (coefficient signs and *p*-values)

Variables (reference)	Potassium Intake			Sodium Intake		
	Q1	Q2	Q3	Q1	Q2	Q3
(Intercept)	0.001	0.000	0.000	0.001	0.000	0.000
Gender (men)	1.000	**(−)0.000**	**(−)0.000**	**(−)0.000**	**(−)0.000**	1.000
Urban area (formal)	0.295	0.155	**(+)0.027**	**(+)0.010**	**(+)0.001**	**(+)0.000**
Age	0.160	0.738	0.430	0.512	**(−)0.042**	**(−)0.026**
Academic qualifications (None and preschool)						
Primary	0.095	0.103	0.214	0.659	0.876	0.256
Secondary	**(+) 0.003**	**(+)0.004**	**(+)0.028**	0.291	0.850	0.274
High school	**(+) 0.000**	**(+)0.000**	**(+)0.000**	1.000	1.000	**(−)0.000**
Professional Status (Employed)						
Unemployed	**(+) 0.050**	0.477	0.437	0.147	0.588	0.068

Q1, 25th percentile; Q2, median; Q3, 75th percentile

Values that are statistically significant at the 5% level are marked in bold

Regarding potassium intake, education level (secondary and high school) seems to have a positive effect on potassium intakes in all quantiles. For Q1, unemployment presented a borderline significance. For Q2 and Q3, gender impacted potassium intake, with a worse situation for women compared to men. Finally, for Q3, the type of urban area had a significant impact on potassium values (*P* = .027).

In terms of the sodium intake, models have shown a significant effect of gender on lower (Q1) and intermediate (Q2) values of sodium, revealing a better situation for women compared to men. The effects of the type of urban areas were significant in all quantiles, with increased values in informal areas. At a significance level of 5%, a unit change of age (quantitative variable) seems to reduce sodium values at Q2 and Q3 levels. Academic qualifications seem to have an effect on Q3. In fact, comparing the high school with none or preschool (reference category), the model indicates a reduction in high sodium intakes for participants with high school levels. The effect of unemployment on sodium intakes was not significant.

## Discussion

Due to the increase of chronic, non-communicable diseases, and the lack of studies done in Cape Verde, it is important to better understand local inhabitant’s dietary habits, particularly with sodium and potassium intakes, and the influence living in different communities has on this habit. This study aimed to estimate potassium and sodium intake, and analyse the self-reported salt intake of men and women, living in formal or informal areas of the city of Praia. Multivariate analyses revealed the important role of these variables in potassium and in sodium intakes.

The Cape Verdean population is a young population. In 2016, official data reported a mean age of 28.3 years and a percentage of 46.3% of the residents between 25 and 64 years old [39]. Particularly in the informal areas, the unemployment rate (27.5% for men and 31.6% for women) was higher than the official unemployment rate (20.0%) in Praia [40].

For women, self-reported hypertension was higher in informal areas (31.0%) than formal areas (19.7%). For men, this self-reported hypertension percentage was 12.2%. The higher percentage of self-reported hypertension among women compared to men may be due to more awareness and monitoring of health status [41–43]. This is also described in a systematic review on hypertension awareness in 26 African countries [44].

Our results on overweight and obesity, with 60.5% of women and 36.9% of men qualifying as such, corroborate the situation described by Ng et al. [45] in Cape Verde, where 59.4% of women (> 20 years) and 38.8% of men (> 20 years) presented overweight and obesity. The WC followed the same trend as the BMI values, higher in women than in men. Similar to other studies in populations of sub-Saharan Africa [46–48], the percentage of women with cardiometabolic risk (based on as WC) was higher than in men.

Despite women presenting higher percentages of hypertension, overweight or obesity, and metabolic risk when compared to men, men presented higher values of daily sodium intake. These results may be explained by the higher median age and lower educational level of women compared to men. Furthermore, the role of women in the family structure and society may help explain this result. It is common for women not to live with the father of their children, receiving little to no emotional and/or financial support. Compared to men, the women carry significant responsibilities and a disproportional social pressure to care for the children [30, 49, 50]. In contrast, our previous study (*n* = 1912) showed that men reported being more physically active, particularly during leisure time [30]. The number of steps recorded in working and non-working days in a subsample (*n* = 118) was higher in men compared to women [30].

Regarding potassium intake, a study involving 18 countries (including South Africa) estimated average potassium excretion at 2120 mg/day [13]. This work indicated that South Africa and certain middle-income countries presented lower excretion values (1700.0 mg/day) [13]. Cohall et al. [51] also described an inadequate potassium intake (2950.0 mg/day) in a sample of Afro-Caribbean’s from Barbados Island. In our study, we found a median potassium intake of 2924.2 mg/day for men and a median of 2562.6 mg/day for women. Almost 70.0% of men and 80.0% of women ingested less than the recommended 3510 mg/day of potassium. In a study carried out in the capital of another Portuguese-speaking country, Maputo, Mozambique, this percentage was 96.0% [52]. In South African adults, the non-compliance with the daily potassium recommendation was 91.0% [53].

Inadequate potassium intake may be related to low consumption of fruits and vegetables. Daily fruit consumption was reported by more than half of participants. However, a daily vegetable consumption was only reported by 28.9% of men and 36.1% of women. In a previous study on Cape Verdean families, a lower consumption of fruits and vegetables was also described [28]. Olack et al. [46] reported an insufficient consumption of fruits and vegetables in 98.8% of participants living in an urban slum in Nairobi.

In a study with 66 countries, including various African countries, the estimated average intake of sodium was 3950.0 mg/day, varying between 2180.0 mg/day and 5510.0 mg/day [3]. With regards to sodium intake in our study, statistical differences were observed by urban areas and by gender. Sodium intake was higher for men living in informal areas (4131.2 mg/day) compared to men living in formal areas (3014.6 mg/day). The median sodium intake for women in the informal area was 3243.4 mg/day, higher than that of women in the formal areas whose median sodium intake was 2522.4 mg/day.

Our values of sodium consumption exceeded the estimates of Noubia et al. [6], which estimated daily sodium intake in sub-Saharan Africa at less than 3300.0 mg/day.

Regional mean intakes in Middle East/North Africa were highest, ranged from 3900.0 to 4200.0 mg/day [9]. Our study showed that 79.9% of women and 70.6% of men are exceeding the WHO recommendations for sodium intake. This percentage of noncompliance with WHO recommendations was larger than that in South Africa (69.0%) [53], but smaller than that in Maputo, Mozambique (92.0%) [52].

Intra-urban comparisons are rare, especially when describing sodium consumption. Some studies compare sodium consumption in urban areas and rural areas and between men and women [21, 53–56]. A systematic review on salt intake in sub-Saharan Africa included 17 studies, 10 of which reported gender differences, with higher values for men [21].

In terms of self-reported salt intake, most of our participants mentioned a “low” or “normal” salt consumption. However, for all self-reported categories, sodium intake values based on 24HDR were concerned. Newson et al. [57] analysed the barriers to progress in reducing salt intake in seven countries, including South Africa. They concluded that 58.0% of participants reported *“never or rarely”* adding salt to food compared to 28.0% who reported doing so *“regularly or ever”.* Moreover, 55.0% of participants reported they did not know the daily recommendation of salt intake [57], and 32.0% reported that they knew daily recommendation, but incorrectly identified it. The study suggested that a lack of knowledge regarding sodium recommendations, and an inability to quantify salt intake are large barriers to improving diet [57].

To our knowledge, very few studies use quantile regression models to analyse the effect of sociodemographic variables on potassium and sodium intakes in African populations. Quantile regression models have revealed the influence of gender and urban area on potassium and sodium intakes, at least in some quartiles. Adjusted models seem to highlight academic qualifications and age as explanatory variables for some quantiles of potassium and sodium intakes, while only a borderline significant effect of unemployment was found in a model for the first quartile of potassium intake. It is important to consider the effects of sociodemographic variables on nutrient intake at different distribution points, not just on the mean effect. Higher sodium intake in younger people may be due to increased consumption of processed foods, rich in salt, that are available throughout the city and consistent with the city’s nutritional transition state [28, 29]. According to multiple regression models from a study in Benin, a positive association between age (younger) and a higher sodium intake was found [56]. For potassium intake, males were positively associated with higher values for sodium [56]. Socioeconomic inequalities were also related to the intake of sodium, potassium and other micronutrients in diverse populations [58–60]. Higher education levels and income were associated with a better diet quality and nutrition profile [58, 61]. The socioeconomic inequities influence the availability, affordability and food preferences [58, 61], and this reinforces the need of more intra-urban studies in Africa.

### Limitations and opportunities

This is a cross-sectional study with self-reported data which may lead to some bias of the information. The use of self-reported hypertension information was used as a strategy to obtain answers from more participants at a lower cost. But this strategy can cause problems, as a lower awareness regarding hypertension has been described in African countries and other low- and middle-income countries [41, 44, 62]. This may be explained by the difficulty of access to health services, or the high rates of illiteracy and poverty [62]. However, it is important to note that the Cape Verdean population has a literacy rate higher than the most sub-Saharan African countries [63]. The city of Praia stands out from the other municipalities in the country with 16.2% of the inhabitants with higher education [40]. Literacy can contribute to a better perception and reporting of hypertension. In other regions, it had been reported that a reasonable or adequate agreement between the reported information and the measurement [41, 64–68]. Therefore, self-reported hypertension data may be a reliable estimate of the disease prevalence of in more developed communities [41].

Fruits and vegetables consumption was obtained from a questionnaire of the frequency of food consumption that presents some well-known limitations [69, 70]. This study only used this questionnaire to explore the frequency of consumptions of these foods groups, and not to estimate nutrients intakes.

The question of self-reported salt intake is a subjective issue. However, this question revealed a disconnection between the perception of salt intake by participants and the values provided by 24HDR. This discrepancy is important to consider when designing interventions to reduce salt consumption.

Dietary intake was collected only with one 24HDR which can limit the accuracy of potassium and sodium intakes. Reporting or quantification errors are common in this method [69–71]. A specific food composition table does not exist in Cape Verde, which may add more bias to the results. The Portuguese Food Composition Table [33] and West African Food Composition Table [34] are used by Cape Verdeans nutritionists. This fact may affect the values of potassium intake, particularly from tropical fruits and vegetables. However, comparisons by gender and by urban areas are not compromised by this issue. Furthermore, this issue reveals the need of a country-specific Food Composition Table in Cape Verde.

The salt added in cooking or added to foods was not measured accurately, which underestimated the values of the sodium intake. Even with these limitations, this study adds to our understanding of this understudied African city. Gender analysis is important to develop dietary guidelines and food labels [2].

## Conclusion

The informal urban areas showed higher vulnerability compared with formal areas, presenting a disadvantageous situation in terms of some sociodemographic characteristics and self-reported chronic diseases (including self-reported hypertension). Overweight or obesity and cardiometabolic risk are more concerning for women.

In our sample, we observed a low potassium intake and a high sodium intake, relative to the WHO recommendations. Potassium intake was statistically lower for women than for men. Sodium intake was higher in informal areas for both sexes and was statistically higher for men compared to women. Sodium intake did not correspond to self-reported salt intake. Through the multivariate analysis, gender and type of urban area seem to impact on potassium and sodium intakes in different quantiles. Findings from intra-urban studies are essential to the design and execution of efficient health education policies related to this double health threat (low potassium intake and a high sodium intake), particularly when considering the specific characteristics of inhabitants in different neighbourhoods and by gender, to effectively reduce the risk of hypertension and other cardiovascular diseases.

## Abbreviations

24HDR: 24-h dietary recall
BMI: Body Mass Index
IQR: Interquartile interval
Q1: 25th percentile
Q2: 50th percentile (median)
Q3: 75th percentile
WC: Waist circumference
WHO: World Health Organization
